# Dr. Maton's Case of Catalepsy

**Published:** 1802-01

**Authors:** W. G. Maton

**Affiliations:** Craven Street


### *To the Editors of the Medical and Physical Journal.*

GENTLEMEN,

THE following case, though it contains no instance of the efficacy of medicine, yet, as it adds to the history of disease, may not prove unacceptable to your readers, to whom its singularity may also render it interesting.

Two or three years ago, a gentleman requested my opinion respecting a peculiar affection to which one of his sons, a delicate little boy, became subject about the 6th year of his age. Notwithstanding the apparent delicacy of his habit, however, I was informed that at the time of his birth he was a very fine, healthy infant, and continued so until he became ten months old, when he was attacked with a cough and fever so violently that his life was despaired of. The cough was declared by the faculty to be the hooping cough. At the age of six, as I have before mentioned, he began to experience seizures of a very remarkable kind, which were thus described by the parent, and which I found afterwards, in consequence of having opportunities of observation myself, to be perfectly correct.

The child was seen suddenly to suspend whatever employment he happened to be engaged in, or whatever attention he was exercising, and to become perfectly insensible to every thing thing that was paſſing around him. Neither expreſſions nor geſtures of any perſon near him, no ſound nor event whatſoever, ſeemed to make any impreſſion on his ſenſes. Whatever attitude, however, he was in before an attack, that attitude he retained. If, for inſtance, he was in the act of drinking, he would continue to hold the glaſs or cup perfectly firm and ſteady, and after the ſeizure ſwallow its contents, as though nothing had happened; if he was ſtanding or walking, he remained firm on his legs, though in the *latter* caſe unable to keep them in motion. When aſked after one of theſe ſeizures what he had felt, he uſed to ſay, that "he had only been in one of his *puzzles*, and that he had felt nothing." The ordinary duration of one of theſe *puzzles*, as the child emphatically termed them, was never leſs than a minute, or longer than two minutes, but they often recurred to the number of twenty or thirty in a day. He ſeldom failed to be attacked at dinner time, and when he was giving extraordinary attention to a book, or to muſic, for the notes in which the child has a moſt exquiſite ear, and for its performance he poſſeſſes moſt ſingular talents. He has alſo a very acute underſtanding, great facility and deſire of acquiring knowledge, and a moſt mild, amiable, and affectionate diſpoſition. The ſeizures were firſt noticed by his writing-maſter, who complained of the child having been very inattentive to his buſineſs. This circumſtance naturally ſurpriſed his parents, on account of his uncommon docility; and when they aſked him why he gave his maſter ſo much trouble, he declared that he could not help it, for that he had of late very often loſt his ſight, and knew not what he was about. They were then induced to obſerve him very attentively, and ſoon diſcovered the malady under which he laboured. Having enquired if they were aware of any event or circumſtance that gave origin to theſe ſingular ſeizures, I was informed that the only one, likely to be connected with the complaint, which had come to their knowledge was the following. The child had by accident ſeen a horſe killed, and felt ſo much horror at the ſpectacle that he became very ſick at the ſtomach, and a violent fluſhing (of a bright ſcarlet colour) overſpread his face and neck, accompanied by unuſual agitation and diſtreſs of mind. Theſe unpleaſant ſymptoms, however, ſoon diſappeared after medical aſſiſtance had been called.

My ideas of the nature of the extraordinary ſeizures was, that they were referable perhaps to a diſeaſe which has been denominated *catalepſy*, but which it has fallen to the lot of very few practitioners to witneſs, and therefore to acquire an accurate knowledge of. With reſpect to medicine, as the child exhibited in all other reſpects the appearance of perfect health, digeſtion being being unimpaired and all the functions natural, I was decidedly averse from its use, for I did not know in what way it was to affect the seat or causes of the complaint. I therefore fairly acknowledged to the parents that I was unacquainted with any remedy, except what the constitution might itself effect during those changes which precede the period of puberty, or which might be produced by the supervention of some acute disease. During my annual visit at Weymouth, the last summer, I received from the father of the young gentleman the agreeable intelligence, that the latter was perfectly free from those distressing symptoms under which he had laboured for nearly four years. In the month of February last he was attacked (whatever may have been the nature of the cough that came on in his infancy) with genuine *pertussis*, which harassed him very severely. When that disease had reached its utmost degree of violence, which was about a month after its first attack, *cataleptic seizures* (if they may be so called) wholly left the child, no traces of them having since been observable.

I am, &c.

*Craven Street,*
*November* 30, 1801.

W. G. MATON.

